# Optimizing chili production in drought stress: combining Zn-quantum dot biochar and proline for improved growth and yield

**DOI:** 10.1038/s41598-024-57204-w

**Published:** 2024-03-19

**Authors:** Misbah Hareem, Subhan Danish, Mahnoor Pervez, Usman Irshad, Shah Fahad, Khadim Dawar, Sulaiman Ali Alharbi, Mohammad Javed Ansari, Rahul Datta

**Affiliations:** 1 Department of Environmental Sciences, Woman University Multan, Multan, Punjab Pakistan; 2 Department of Soil Science, Faculty of Agricultural Sciences and Technology, Bahauddin Zakariya University, Multan, Punjab Pakistan; 3 Department of Zoology, Lahore College for Women University, Lahore, Pakistan; 4 Department of Environmental Sciences, COMSATS University Islamabad Abbottabad Campus, Abbottabad, Pakistan; 5 Department of Agronomy, Abdul Wali Khan University Mardan, Khyber Pakhtunkhwa, 23200 Pakistan; 6 Department of Natural Sciences, Lebanese American University, Byblos, Lebanon; 7 Department of Soil and Environmental Science, The University of Agriculture Peshawar, Peshawar, Pakistan; 8 Department of Botany and Microbiology, College of Science, King Saud University, PO Box -2455, 11451 Riyadh, Saudi Arabia; 9 Department of Botany, Hindu College Moradabad (Mahatma Jyotiba Phule Rohilkhand University Bareilly), Moradabad, India; 10 Department of Geology and Pedology, Faculty of Forestry and Wood Technology, Mendel University in Brno, Zemedelska 1, 61300 Brno, Czech Republic

**Keywords:** Antioxidant, Biochar, Chlorophyll content, Chili, Drought, Morphological attributes, Proline, Plant sciences, Plant stress responses, Drought

## Abstract

The reduction in crop productivity due to drought stress, is a major concern in agriculture. Drought stress usually disrupts photosynthesis by triggering oxidative stress and generating reactive oxygen species (ROS). The use of zinc-quantum dot biochar (ZQDB) and proline (Pro) can be effective techniques to overcome this issue. Biochar has the potential to improve the water use efficiency while proline can play an imperative role in minimization of adverse impacts of ROS Proline, functioning as an osmotic protector, efficiently mitigates the adverse effects of heavy metals on plants by maintaining cellular structure, scavenging free radicals, and ensuring the stability of cellular integrity. That’s why current study explored the impact of ZQDB and proline on chili growth under drought stress. Four treatments, i.e., control, 0.4%ZQDB, 0.1 mM Pro, and 0.4%ZQDB + Pro, were applied in 4 replications following the complete randomized design. Results exhibited that 0.4%ZQDB + Pro caused an increases in chili plant dry weight (29.28%), plant height (28.12%), fruit length (29.20%), fruit girth (59.81%), and fruit yield (55.78%) over control under drought stress. A significant increment in chlorophyll a (18.97%), chlorophyll b (49.02%), and total chlorophyll (26.67%), compared to control under drought stress, confirmed the effectiveness of 0.4%ZQDB + Pro. Furthermore, improvement in leaves N, P, and K concentration over control validated the efficacy of 0.4%ZQDB + Pro against drought stress. In conclusion, 0.4%ZQDB + Pro can mitigate drought stress in chili. More investigations are suggested to declare 0.4%ZQDB + Pro as promising amendment for mitigation of drought stress in other crops as well under changing climatic situations.

## Introduction

In recent times, drought stress has emerged as a primary environmental challenge, significantly impeding plant growth^1^. When plants face drought stress, their roots encounter difficulties in absorbing sufficient water, which consequently increases the rate of transpiration^2^. The impacts of drought stress are manifold, encompassing compromised growth, reduced yield, diminished membrane integrity, altered pigment content, disruptions in osmotic adjustment water relations, and impaired photosynthetic activity^3^. In response to water scarcity, plants employ various mechanisms to adapt, leading to adaptive changes in growth patterns and physiological-biochemical processes. These adaptations include modifications in plant structure, adjustments in growth rates, alterations in tissue osmotic potential, and enhancement of antioxidant defenses^4^. To overcome this issue use of proline, biochar and zinc (Zn) quantum dots are becoming popular.

Biochar has porous structure that significantly boosts water retention in soil, ensuring a steady supply for plant absorption^5–11^. It also aids in preserving and enhancing nutrient availability and decreasing the impact of abiotic stress^9,10,12–14^. Furthermore, better uptake of nutrients via biochar application also promotes the chlorophyll contents and improve gas exchange attributes under stress condition^14^. Research has also indicated that quantum dots (QDs) possess the capability to improve plant growth and alleviate the impacts of oxidative stress by regulating functioning of antioxidants^15^. Additionally, the ZnO QDs also promote the uptake and translocation of nutrients which in turn enhance plant biomass^8,15^.

Proline, a crucial amino acid for plants under stress from drought, extreme temperatures, and salinity, serves as an osmoprotectant, safeguarding against dehydration by accumulating in cells^16^. Its antioxidant properties shield against oxidative damage caused by stressors like drought. Proline role in stabilizing proteins and membranes maintains cellular integrity^17^, while its influence on cellular balance and gene expression underscores its significance in facilitating plant growth amid tough conditions^18,19^.

Chilies are renowned for their remarkable versatility, finding applications in culinary, medicinal, and agricultural domains owing to their abundant content of capsaicinoids, vitamins, carotenoids, and minerals^20^. Heir culinary significance transcends geographical boundaries, enriching global dishes while offering potential health benefits through their antioxidant properties^21^. Nevertheless, chili farming encounters significant hurdles, particularly from drought stress. Inadequate water supply during crucial growth stages detrimentally impacts plant development, resulting in smaller fruits, diminished yield, and increased vulnerability to diseases and pests. Prolonged drought exacerbates these challenges by impairing the plant's capacity to synthesize essential compounds such as capsaicinoids and vitamins, thereby compromising the quality and quantity of chili harvests^22^.

That’s why current study aims to explore the potential of proline foliar and zinc-quantum dot biochar (ZQDB) on chili plants cultivated in drought stress. This study is covering the knowledge gap regarding the use of proline and ZQDB as combined treatment to alleviate the drought stress in chili. The novelty of the current study lies in the combined application of proline and ZQDB for mitigating drought stress. It is hypothesized that applying proline and ZQDB might be effect technique to mitigate the adverse effects of drought stress on chili plants, potentially enhancing their growth and productivity.

## Material and methods

### Experimental site

**Figure 1:**
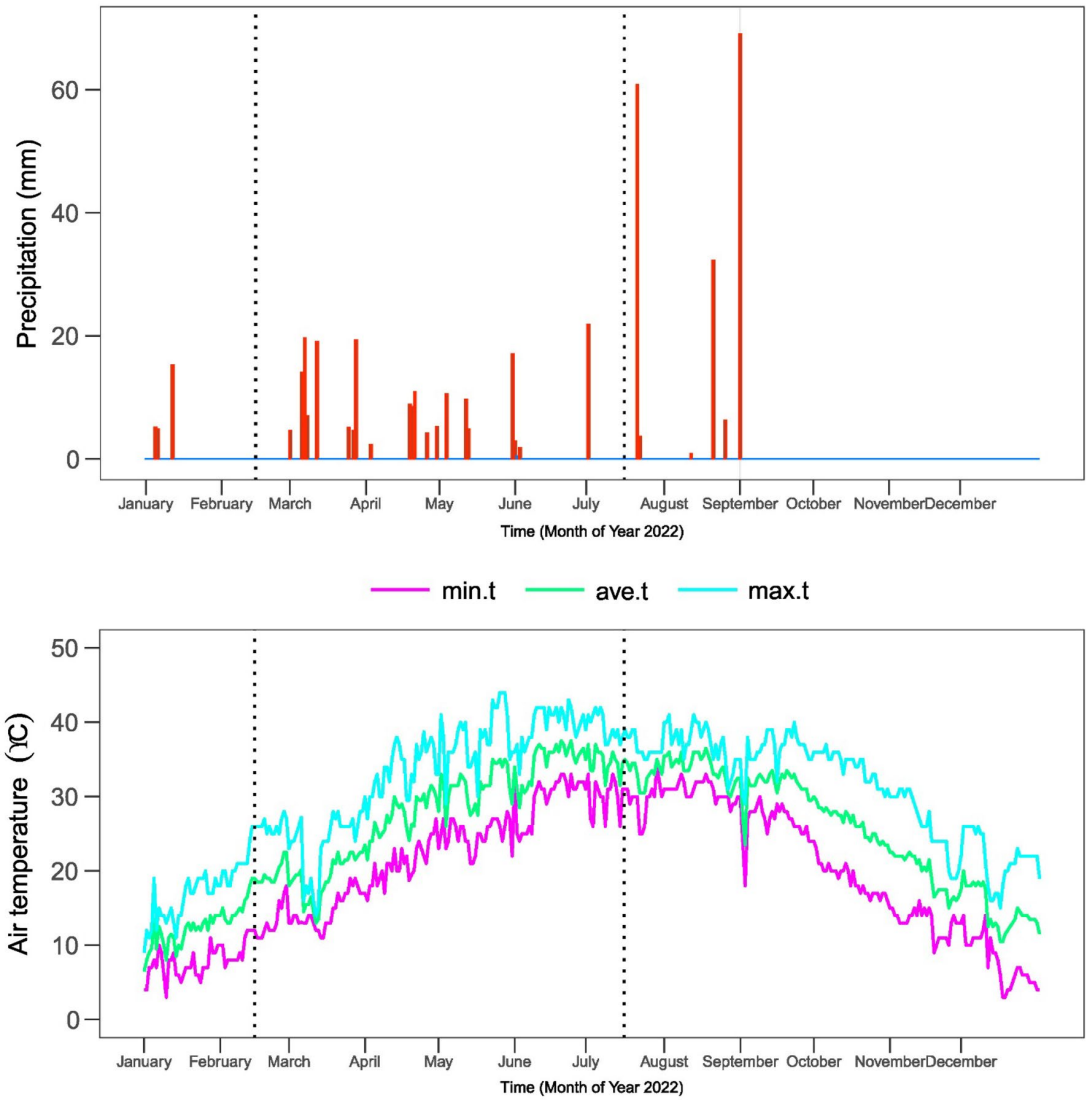
Climatic data of experiment.

In 2022, experimental research was carried out in research area of ResearchSolution, situated in Multan, Punjab, Pakistan. The research site's geographical coordinates are 30°15′49″N and 71°30′35″E. The climatic data of the experiment is provided in Fig. 1.

### Zn-quantum dots biochar (ZQDB)

For synthesis of zinc quantum dots (ZQD) standard protocol was adopted as described by^8^. For the production of biochar, cabbage waste obtained from the local market at coordinates 30°11′29.8"N 71°28′48.8"E was collected. The collected waste underwent initial sun-drying before undergoing pyrolysis under partially aerobic conditions at a temperature of 325 ± 5 °C. The characteristic of pre-experimental biochar is given in Table 1. This mixture of biochar and ZQD (99:1) underwent stirring for 24 h to facilitate the binding of quantum dots with biochar. Following this period, the ZQDB mixture was subjected to multiple washes with ethanol to eliminate unbound quantum dots. Subsequently, the quantum dots biochar blend underwent drying in a vacuum oven at 60 °C for 24 h.

**Table 1:** Pre-experimental soil, biochar, and irrigation characteristics.

Soil	Values	Biochar	Values	Irrigation	Values
SOC (%)	0.41	pH*s*	6.93	Carbonates (meq./L)	0.001
EP (mg/kg)	9.28	EC*e* (dS/m)	3.39	Chloride (meq./L)	0.13
TN (%)	0.0025	Ash Content (%)	32.99	Bicarbonates (meq./L)	4.35
AK (mg/kg)	132	Volatile Matter (%)	17.79	Sodium (meq./L)	31.96
pH*s*	9.01	Fixed carbon (%)	47.99	Ca + Mg (meq./L)	4.05
EC*e* (dS/m)	1.97	TP (%)	0.69	EC (µS/cm)	543
Sand (%)	28	TN (%)	1.37	pH	6.50
Silt (%)	38	TK (%)	3.25	TN = Total Nitrogen EP = Extractable Phosphorus AK = Available Potassium CEC = Cation Exchange Capacity EC = Electrical Conductivity	
Clay (%)	37	CEC (meq./100 g)	403		
Texture	Clay Loam	Surface area (m^2^/g)	320		

### Collecting, sterilization, and sowing of seeds

The chili seeds utilized were obtained from a licensed seed dealer authorized by the Government of Punjab, Pakistan. Before sowing, 5% sodium hypochlorite solution was used for sterilization. Each pot containing 5 kg of soil (the physicochemical attribute of pre-experimental soil is provided in Table 1) was initially seeded with 15 seeds. Post-germination, a careful thinning process was executed, resulting in the retention of 4 seedlings per pot^23^.

### Fertilizer application

The application included N, P, and K at a rate of 58: 25: 25 kg/acre (0.15: 0.06: 0.06 g/pot) to fulfill the macronutrient needs. Urea, single superphosphate, and potassium sulfate fertilizers were utilized to fulfill the requirement of N, P and K.

### Drought

To simulate conditions of normal soil moisture (No Drought = 65% FC) and drought stress (35% FC), the trial involved manipulating soil moisture levels using moisture meter (YIERYI 4 in 1; Shenzhen, Guangdong Province, China), adhering to a methodology suggested by^24^.

### Treatment plan

There were 2 factors i.e., drought stress and treatments. Total four treatments i.e., control (no proline and no ZQDB), 0.4% zinc quantum dot biochar (ZQDB), Proline (0.1 mM proline), 0.4%ZQDB + Proline (Pro) were applied in four replicates under normal moisture and drought stress following completely randomized design (CRD).

### Growth attributes data collection

Harvesting was done in mid of august. Soon after harvesting data was collected for total plant dry weight (g/plant), plant height (cm), the number of primary branches per plant, fruit length (cm), fruit girth (cm), fruit yield (kg/plant), and chlorophyll content. For dry weight analytical weight balance was used.

### Chlorophyll content

For chlorophyll assessment 0.5 g of freshly harvested leaf samples were grinded in a pestle mortar with 20 ml of 80% acetone. Afterward, the resulting mixture underwent centrifugation at 3000 rpm for 15 min and absorbance was taken at 645 and 663 nm^25^.

$${\text{Chlorophyll a }}\left( {\frac{{{\text{mg}}}}{{\text{g}}}} \right) = \frac{{\left( {12.7{ } \times {\text{ A}}663} \right){ }{-}{ }\left( {2.69{ } \times {\text{ A}}645} \right) \times {\text{V}}}}{{1000{ } \times {\text{W}}}}$$

$${\text{Chlorophyll b }}\left( {\frac{{{\text{mg}}}}{{\text{g}}}} \right) = \frac{{\left( {22.9{ } \times {\text{ A}}645} \right){ }{-}{ }\left( {4.68{ } \times {\text{ A}}663} \right) \times {\text{V}}}}{{1000{ } \times {\text{W}}}}$$

$$\mathrm{Total Chlorophyll }\left(\frac{{\text{mg}}}{{\text{g}}}\right)=\frac{20.2\left({\text{A}}645\right)+8.02\left({\text{A}}663\right)\times {\text{V}}}{1000 \times {\text{W}}}$$

### Antioxidant assays

To assess SOD activity, nitro blue tetrazolium (NBT) was used as per standard protocol. The absorbance reading was taken at 560 nm^26^. For CAT activity enzymatic breakdown of hydrogen peroxide (H_2_O_2_) was assessed at 240 nm^27^. For APX activity, the reation among ascorbic acid and H_2_O_2_ was observed at 290 nm wavelength^28^. The quantification of malondialdehyde (MDA) content was done using thiobarbituric acid method^29^.

### DPPH activity and total phenolic content

The DPPH radical scavenging activity assessment followed the method^30^ employing the artificial 2,2-diphenyl-1-picrylhydrazyl radical (DPPH). Total phenolic content was determined using a modified Folin–Ciocalteu colorimetric method^31^.

### Electrolyte leakage

Uniform leaf sections having weight one gram were placed in a test tube having 20 ml of deionized water. The test tubes were then kept at a consistent temperature of 25 °C for 24 h and solution electrical conductivity (EC1) was measured using a calibrated EC meter. Following this the test tubes were again heated at 120 °C for 20 min in water bath and secdnf electrical conductivity measurement (EC2) was recorded ^32^.

$$\mathrm{Electrolyte \;Leakage }\left(\mathrm{\%}\right)=\left(\frac{{\text{EC}}1}{{\text{EC}}2}\right)\times 100$$

### Fruit harvest, dry weight, and nutrient analysis

In our study, we implemented a methodology involving randomly selecting three plants for each set of replicates, which were subsequently divided into leaves, stems, and roots. These segmented plant parts underwent drying in an oven at 70 ± 8 °C for two days to establish their dry weights and elemental concentrations. All analyses of nutrients were conducted based on the dry-weight measurements.

### N, P, and K leaves

In this study, the determination of nitrogen content followed a modified micro-Kjeldahl method described in previous research^33^. Potassium content analysis utilized a flame photometer connected to a continuous-flow system, specifically employing the microflow automated continuous-flow analyzer III from Italy. Phosphorus content quantification at 420 nm was conducted using a spectrophotometer based on the yellow color method, following procedures outlined in earlier work^34^.

### Statistical analysis

The data was analyzed using conventional statistical methods^35^. The application of a two-way ANOVA was conducted using OriginPro software. Subsequent paired comparisons, graph generation, and principal component analysis were performed using OriginPro software^36^.

### Ethics approval and consent to participate

We all declare that manuscript reporting studies do not involve any human participants, human data, or human tissue. So, it is not applicable. Study protocol must comply with relevant institutional, national, and international guidelines and legislation. Our experiment follows the with relevant institutional, national, and international guidelines and legislation

## Results

### Plant height, dry weight, and number of primary branches/plant

**Figure 2:**
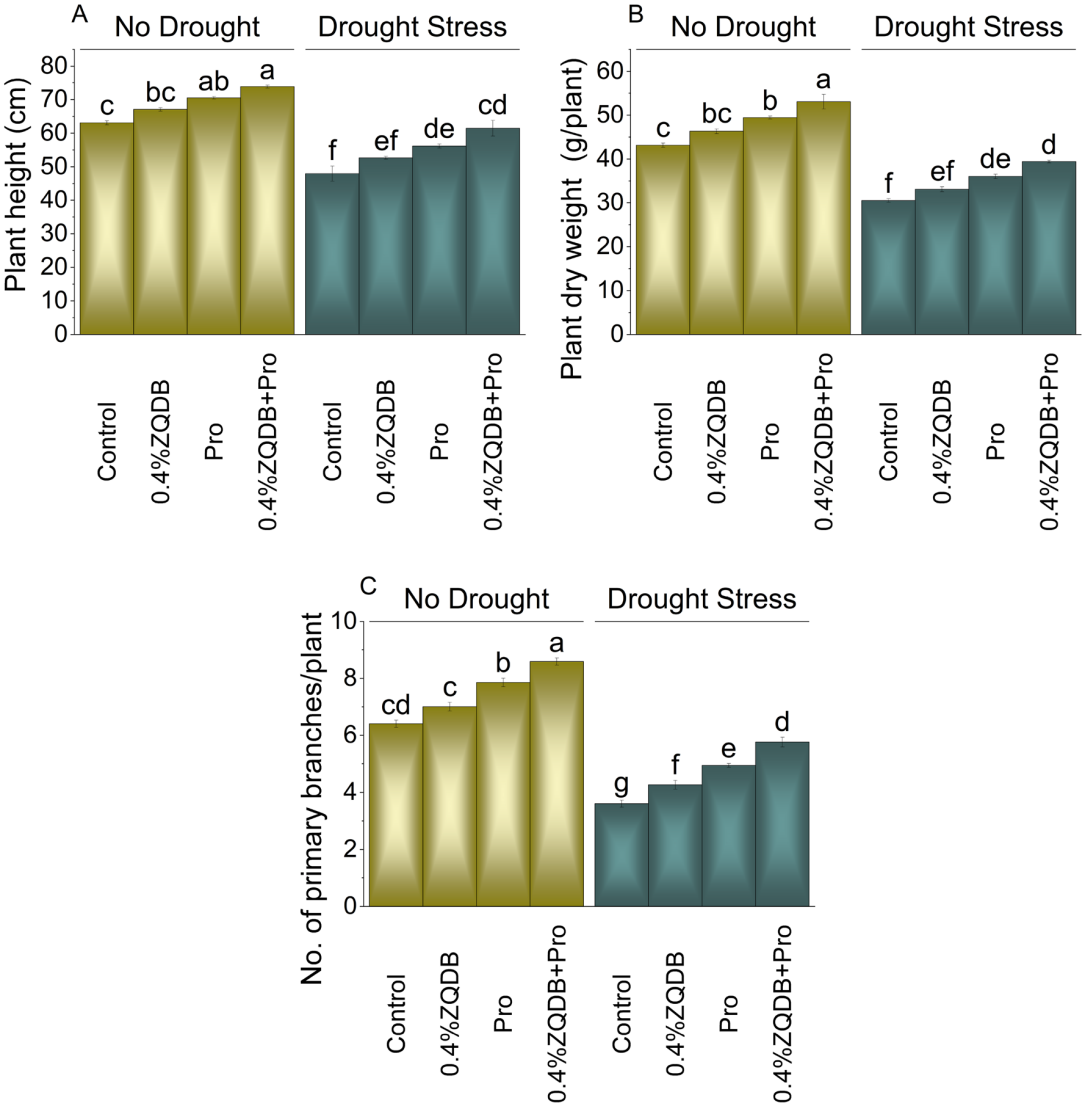
Influence of ZQDB and proline on plant height (**A**), plant dry weight (**B**), and no. of primary branches/plant (**C**) of chili cultivated under no drought and drought stress. The bars represent the mean of four replicates with standard error. The Tukey test revealed significant changes at *p* < 0.05, shown by the different letters on the bars.

Under no drought stress (DS), the addition of 0.4% zinc quantum dots biochar (ZQDB) treatment showed a 6.30% increase in plant height, while the proline (Pro) treatment resulted in an 11.79% increase in contrast to the control. The combined treatment of 0.4%ZQDB + Pro exhibited a 17.09% increase in plant height compared to control under no DS. Under DS, 0.4%ZQDB showed a 9.77%, Pro treatment 17.08%, and 0.4%ZQDB + Pro resulted in 28.12% increase in plant height over control (Fig. 2A).

In case of no DS, adding 0.4%ZQDB resulted in 7.36% increase in plant dry weight over the control. Treatment Pro caused 14.57% and 0.4%ZQDB + Pro showed 22.95% increase in plant dry weight than control under no DS. The application of 0.4%ZQDB, Pro and 0.4%ZQDB + Pro caused 8.60, 18.24 and 29.28% increase in plant dry weight compared to control respectively (Fig. 2B).

The 0.4%ZQDB showed 9.49%, Pro 22.73% and 0.4%ZQDB + Pro 34.16% enhancement than control in no. of branches per plant at no DS. Under DS, applying 0.4%ZQDB, Pro, and 0.4%ZQDB + Pro showed an 18.28%, 37.04%, and 59.80% improvement from control in no. of branches per plant respectively under DS (Fig. 2C).

### Fruit length, girth, and yield

**Figure 3:**
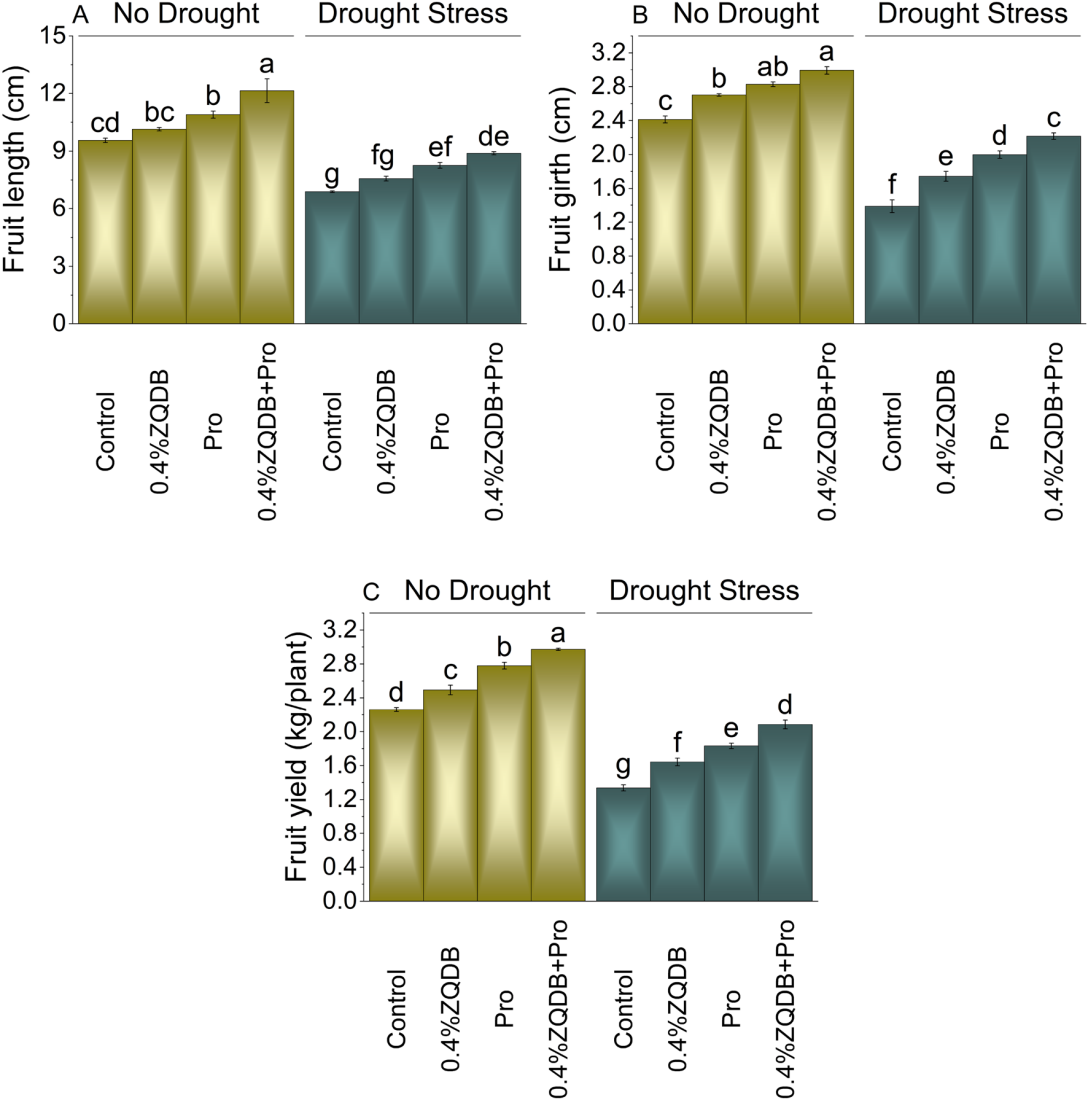
Influence of ZQDB and proline on fruit length (**A**), fruit girth (**B**), and fruit yield (**C**) of chili cultivated under no drought and drought stress. The bars represent the mean of four replicates with standard error. The Tukey test revealed significant changes at *p* < 0.05, shown by the different letters on the bars.

Results showed that 6.07, 14.01 and 27.00% enhancement in fruit length was observed where 0.4%ZQDB, Pro and 0.4%ZQDB + Pro were applied over control under no DS. In DS, 0.4%ZQDB showed 10.05%, Pro 20.06%, and 0.4%ZQDB + Pro 29.20% increase in fruit length than control (Fig. 3A).

Without DS, applying 0.4%ZQDB, Pro and 0.4%ZQDB + Pro caused improvement in fruit i.e., 11.94%, 17.17% and 24.01% compared to control. A significant enhancement of 25.70, 44.07 and 59.81% in fruit girth was noted in 0.4%ZQDB, Pro and 0.4%ZQDB + Pro from control under DS (Fig. 3B).

For fruit yield, 0.4%ZQDB (10.27%), Pro (22.93%) and 0.4%ZQDB + Pro (31.43%) caused enhancement over control under no DS. In case of DS, 0.4%ZQDB exhibited 22.81% while Pro and 0.4%ZQDB + Pro showed 36.90% and 55.78% increase over control (Fig. 3C).

### Chlorophyll content and electrolyte leakage (EL)

**Figure 4:**
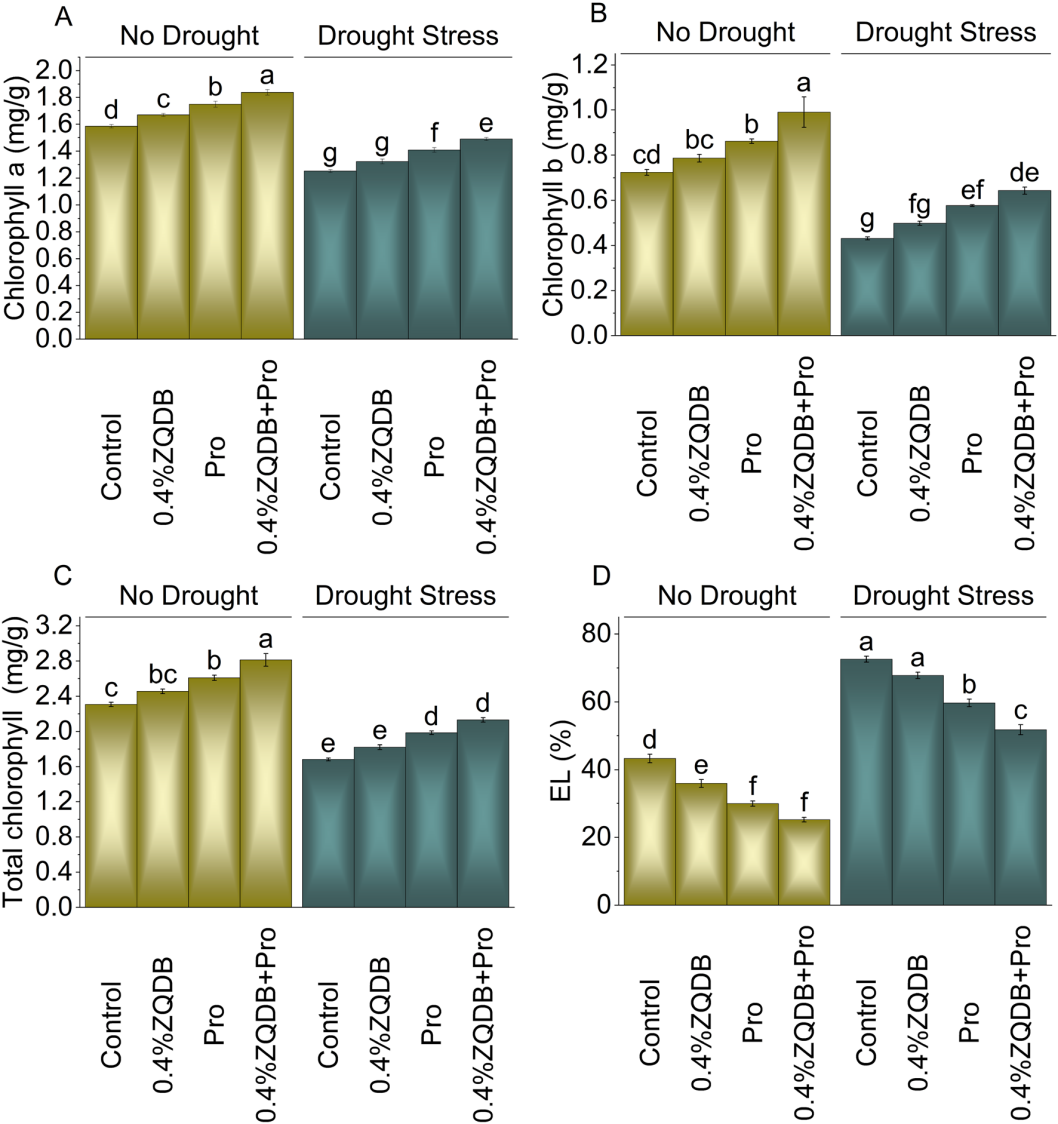
Influence of ZQDB and proline on chlorophyll a (**A**), chlorophyll b (**B**), total chlorophyll (**C**), and electrolyte leakage (EL) (**D**) of chili cultivated under no drought and drought stress. The bars represent the mean of four replicates with standard error. The Tukey test revealed significant changes at *p* < 0.05, shown by the different letters on the bars.

Adding 0.4% ZQDB, Pro, and 0.4% ZQDB + Pro treatment under no drought stress resulted in an increased chlorophyll a (5.36%, 10.35%, and 15.99%), chlorophyll b (8.67%, 19.03%, and 36.82%), and total chlorophyll content (6.40%, 13.07%, and 21.87%) over the control. Introducing 0.4% ZQDB, Pro, and 0.4% ZQDB + Pro led to a 5.67%, 12.50%, and 18.97% increase in chlorophyll a, 15.47%, 33.66%, and 49.02% in chlorophyll b, and 8.18%, 17.92%, and 26.67% in total chlorophyll content in comparison to the control under drought stress (Fig. 4A, B, and C).

In no drought stress, the application of 0.4%ZQDB, Pro, and 0.4%ZQDB + Pro resulted in a 20.51%, 44.48%, and 71.68% decrease in EL compared to the control, and under drought stress, these treatments showed a 7.05%, 21.59%, and 40.17% decrease in EL over the control (Fig. 4D).

### Antioxidant activity (H_2_O_2_, MDA, SOD, and APX)

**Figure 5:**
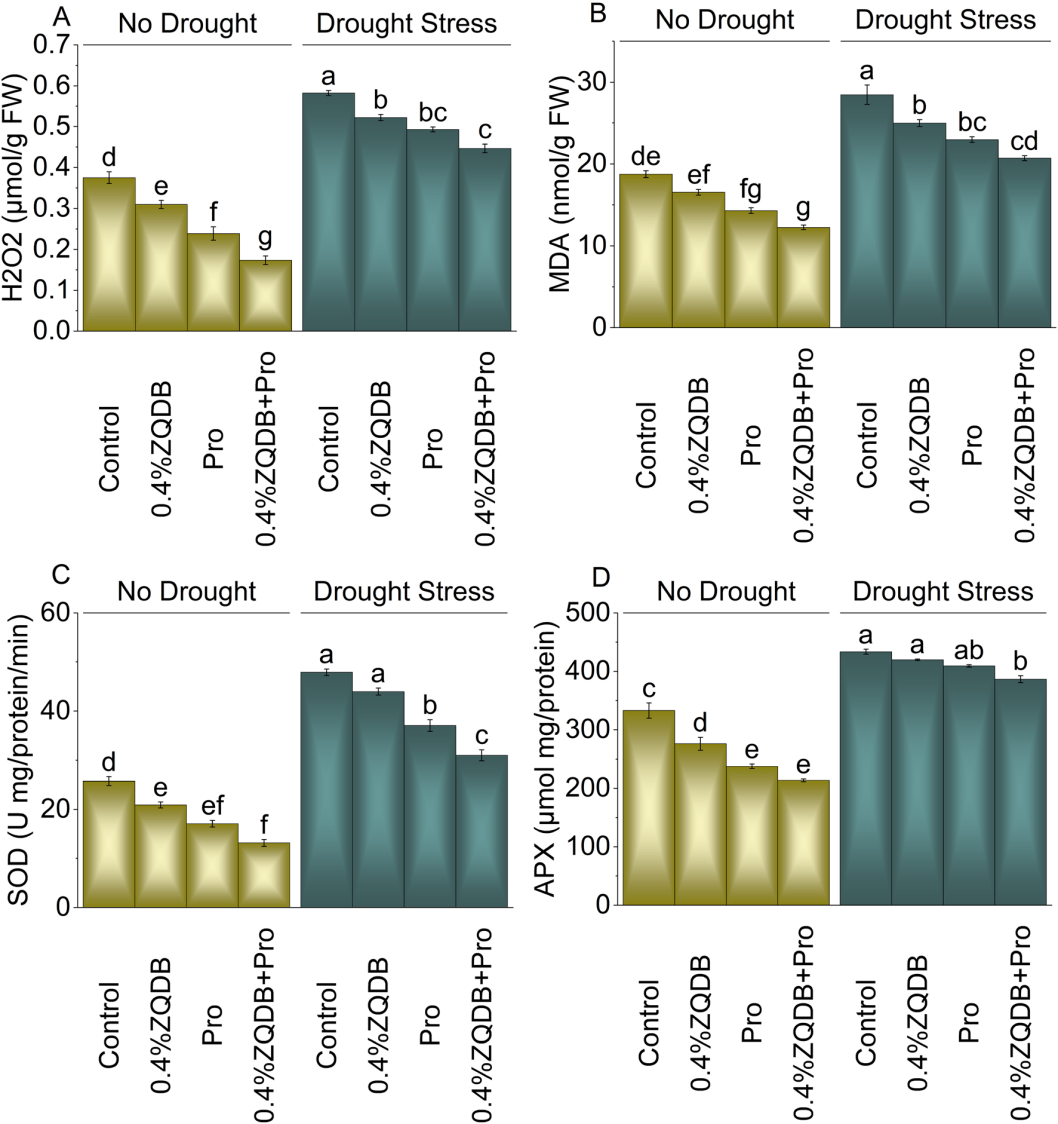
Influence of ZQDB and proline on hydrogen peroxide (H_2_O_2_) (**A**), malondialdehyde (MDA) (**B**), superoxide dismutase (SOD) (**C**), and ascorbate peroxidase (APX) (D) of chili cultivated under no drought and drought stress. The bars represent the mean of four replicates with standard error. The Tukey test revealed significant changes at p < 0.05, shown by the different letters on the bars.

Under no drought stress, the application of 0.4%ZQDB, Pro, and 0.4%ZQDB + Pro treatment showed a decrease in H_2_O_2_ (21.15%, 57.17%, and 115.97%), MDA (13.35%, 31.08%, and 53.04%), SOD (23.13%, 50.54%, and 95.97%), and APX (20.51%, 40.06%, and 55.80%) levels in comparison to the control. Adding 0.4%ZQDB, Pro, and 0.4%ZQDB + Pro treatment in drought stress resulted in a decrease in H_2_O_2_ (11.54%, 18.10%, and 30.33%), MDA (13.96%, 23.95%, and 37.48%), SOD (8.91%, 29.23%, and 54.36%), and APX (3.29%, 5.96%, and 12.15%) level than the control (Fig. 5A, B, C, and D).

### Total phenols, catalase, and DPPH activity

**Figure 6:**
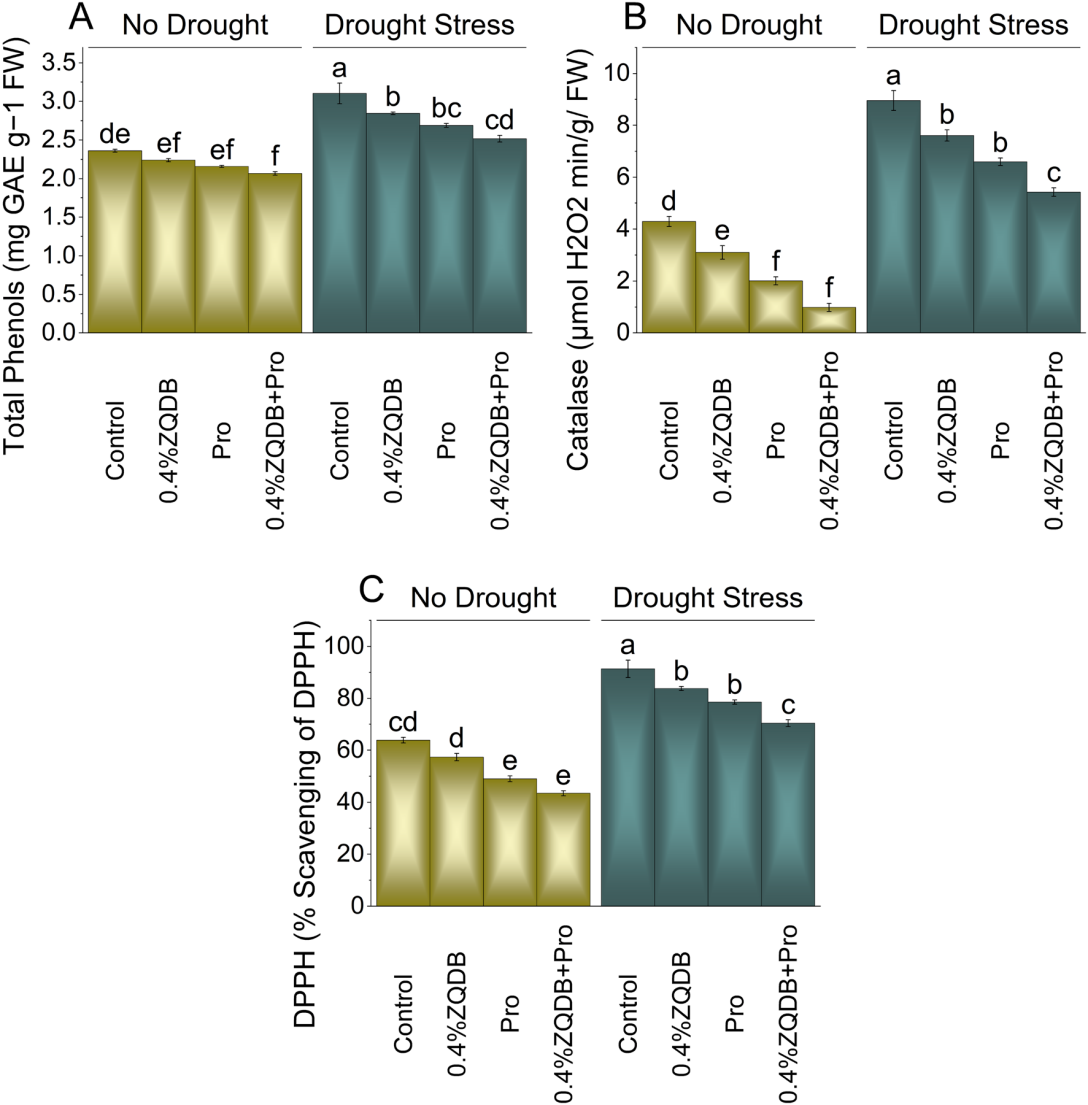
Influence of ZQDB and proline on total phenols (**A**), Catalase (**B**), and DPPH (**C**) of chili cultivated under no drought and drought stress. The bars represent the mean of four replicates with standard error. The Tukey test revealed significant changes at *p* < 0.05, shown by the different letters on the bars.

In no drought stress, 5.46%, 9.39%, and 14.16% decreases were observed in total phenols, 38.28%, 113.43%, and 334.43% decrease in CAT activity and 11.35%, 30.30%, and 46.88% decrease in DPPH activity with the application of 0.4% ZQDB, Pro, and 0.4% ZQDB + Pro treatments over the control. Under drought stress 0.4% ZQDB, Pro, and 0.4% ZQDB + Pro treatments exhibit 9.07%, 15.40%, and 23.29% decrease in total phenols, 17.68%, 35.70%, and 64.87% decrease in CAT activity, and 8.98%,16.23%, and 29.80% drop in DPPH activity compared to the control (Fig. 6A, B, and C).

### Leave N, P, K, and Na

**Figure 7:**
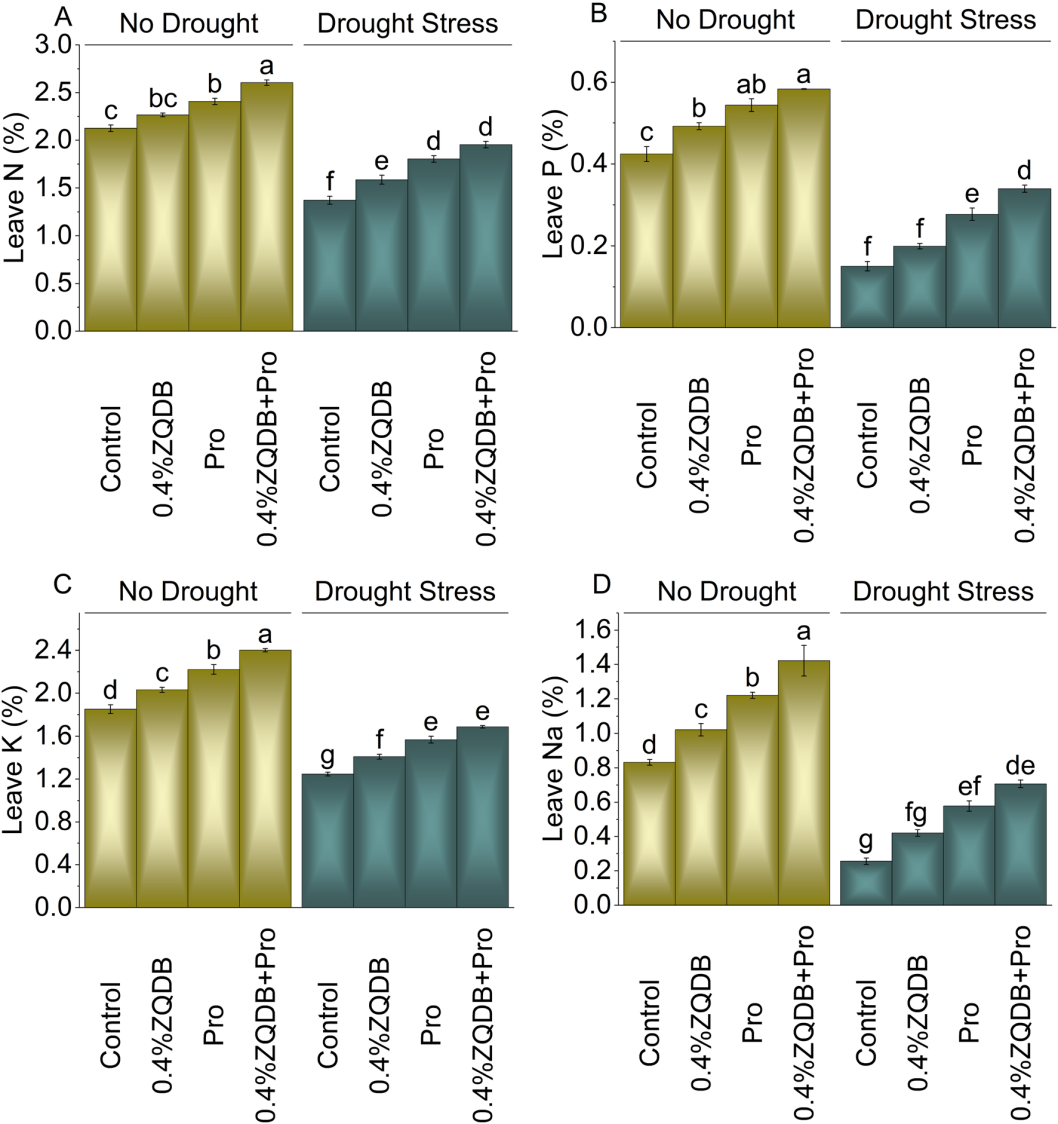
Influence of ZQDB and proline on leave N (**A**), leave P (**B**), Leave K (**C**), and leave Na (**D**) of chili cultivated under no drought and drought stress. The bars represent the mean of four replicates with standard error. The Tukey test revealed significant changes at *p* < 0.05, shown by the different letters on the bars.

The introduction of 0.4%ZQDB, pro, and 0.4%ZQDB + Pro treatment under no drought showed an increase in leave N (6.60%, 13.22%, and 22.60%), leave P (15.97%, 28.11%, and 37.54%), leave K (9.72%, 19.98%, and 29.66%), and leave Na (22.76%, 46.81%, and 71.02%) from the control. However, when subjected to drought stress, the 0.4%ZQDB, pro, and 0.4%ZQDB + Pro treatments showed improvement in leave N (15.63%, 31.52%, and 42.44%), leave P (32.39%, 84.22%, and 125.58%), leave K (12.97%, 25.73%, and 35.29%), and leave Na (64.42%, 125.90%, and 176.15%) in comparison to the control (Fig. 7A, B, C, and D).

### Convex hull and hierarchical cluster analysis

**Figure 8:**
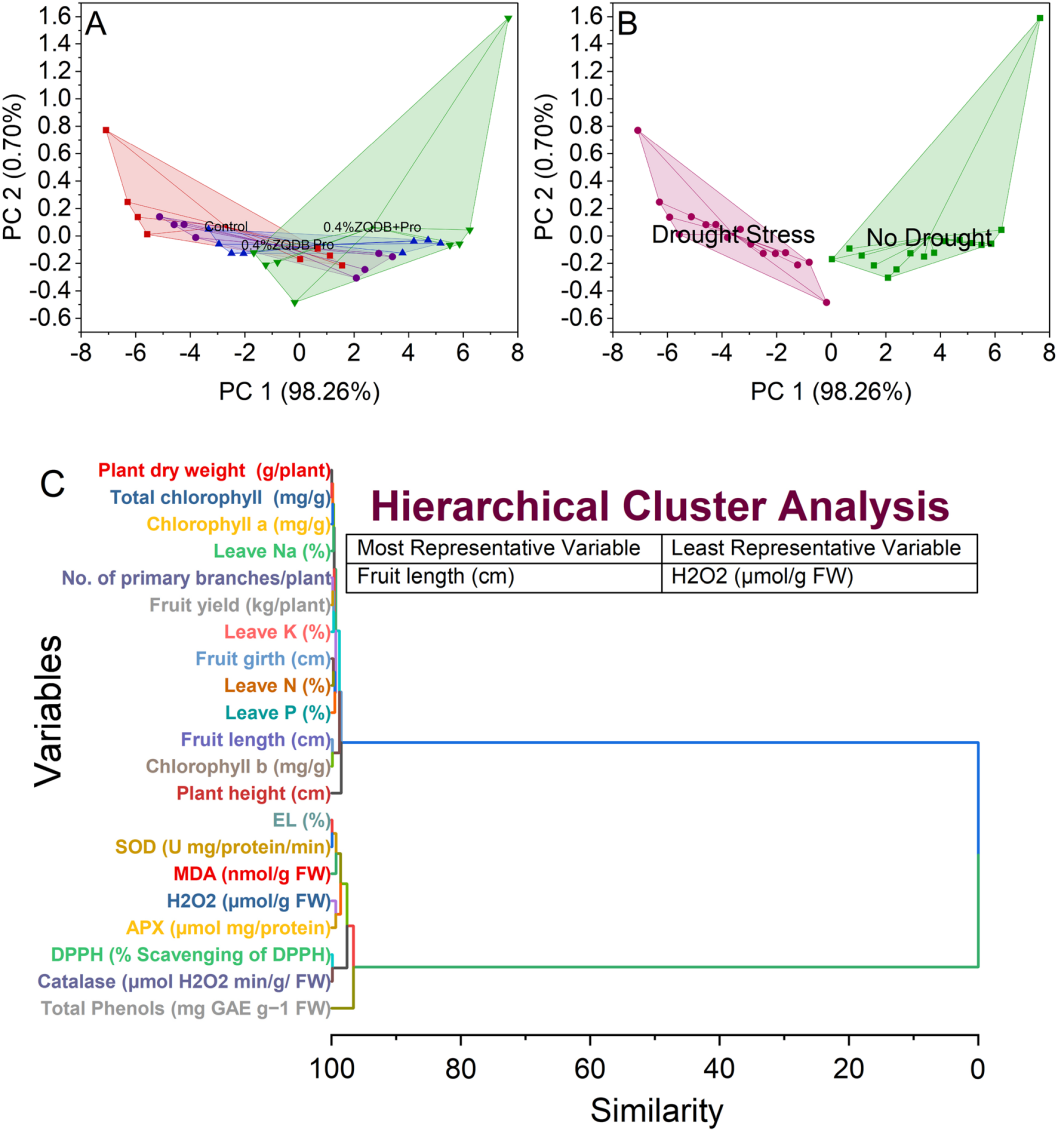
Cluster plot convex hull for treatments (**A**), drought levels (**B**), and hierarchical cluster plot (**C**) for studied attributes.

The control group, 0.4%ZQDB treatment, Pro treatment, and the combined 0.4%ZQDB + Pro treatment occupy their region on the plot, indicating differences in how these treatments affect the variables represented by PC 1 and PC 2. The combined treatment stands out as it forms a centralized cluster, suggesting a distinct effect that differs from individual treatments and the control group (Fig. 8A).

The convex hull analysis was conducted on the dataset based on PC 1 and PC 2, explaining 98.26% and 0.70% of the variation, respectively. The stress distribution indicated two distinct clusters: No Drought and Drought Stress. Convex Hull identified a clear separation between these clusters based on their scores in PC 1 and PC 2. The no drought cluster exhibited scores ranging from 0.02774 to 7.65504 in PC 1 and from − 0.30565 to 1.58993 in PC 2. Meanwhile, the drought stress cluster had scores ranging from − 7.08654 to − 0.17159 in PC 1 and from − 0.48373 to 0.77093 in PC 2. The Convex Hull method delineated the boundary encompassing these distinct groups, illustrating the pronounced separation between the samples experiencing no drought and those under drought stress based on their PC 1 and PC 2 scores (Fig. 8B).

The hierarchical cluster analysis was performed on variables, revealing distinct similarity linkages between various attributes. The analysis identified several clusters based on the similarity in their characteristics. Notably, the variables related to plant physiological traits formed control groups. For instance, Plant dry weight and total chlorophyll exhibited a similarity of 0.07143, indicating a close association between these parameters. Similarly, EL and SOD shared a similarity of 0.09983, suggesting a correlation in their responses.

Further, traits such as fruit length and chlorophyll b displayed a similarity of 0.16232, indicating a relationship between these attributes. Additionally, parameters like no. of primary branches/plant and fruit yield showed a similarity of 0.19166, hinting at a potential connection in their impact on plant productivity. Variables such as fruit girth and leave N exhibited a similarity of 0.30958, indicating a possible relationship between these traits. Chlorophyll a and leave K were similar to 0.21396 and 0.32587, respectively, pointing towards potential interdependencies between these physiological attributes. Interestingly, leave Na and leave P showed a similarity of 0.32657 and 0.54553, possibly suggesting distinct elemental responses within the plant system. The analysis also revealed strong associations within specific physiological attributes, such as H_2_O_2_ and APX, displaying a high similarity of 0.66736, indicative of a close relationship in their responses. Moreover, the hierarchical clustering identified a distinct group comprising plant height, showing a significant similarity of 1.52116, implying a unique attribute set apart from the other variables analyzed (Fig. 8C).

### Pearson correlation analysis

The correlation analysis revealed strong positive relationships among several plant traits. For instance, variables such as plant dry weight and total chlorophyll exhibited a notably high correlation of 0.99859, indicating a close positive association between these attributes. Similarly, attributes like no. of primary branches/plant and fruit yield displayed a strong positive correlation of 0.99621, suggesting a close relationship between the number of branches and fruit yield. Other variables, such as chlorophyll a and b, demonstrated a high positive correlation of 0.99323, indicating a closely linked behavior between these chlorophyll types. Additionally, fruit length and girth showed a strong positive correlation of 0.97541, signifying a relationship between fruit size characteristics.

**Figure 9:**
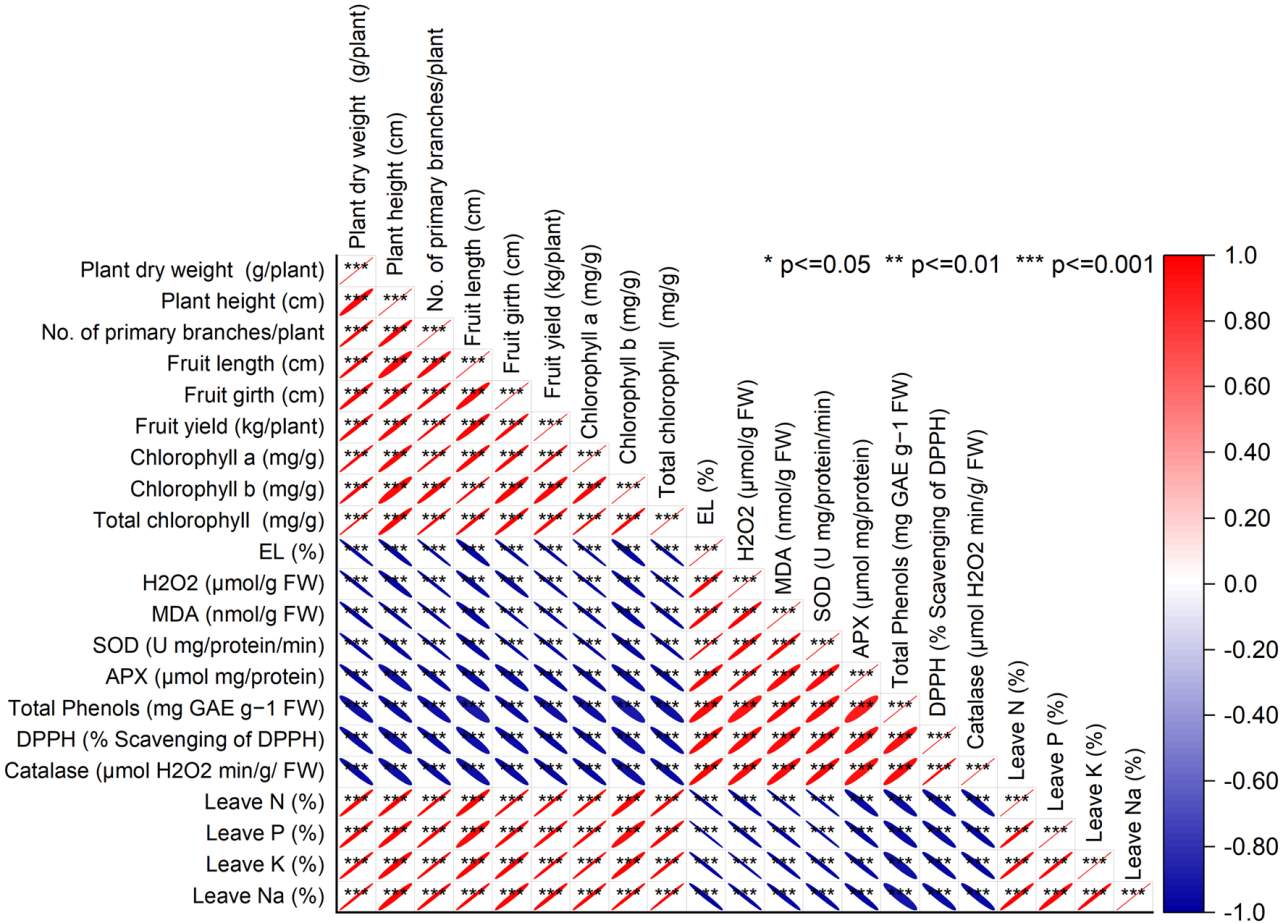
Pearson correlation for studied attributes.

Conversely, specific attributes displayed strong negative correlations. For instance, EL and H_2_O_2_ showcased a substantial negative correlation of − 0.99375, suggesting an inverse relationship between electrolyte leakage and hydrogen peroxide levels. Similarly, leave N and leave P exhibited a negative correlation of − 0.99695, indicating a negative association between the levels of nitrogen and phosphorus in leaves (Fig. 9).

## Discussion

### Drought stress

Drought-induced stress is a prominent abiotic factor impacting crops, initiating biochemical alterations. It significantly hinder plant growth, delay development, and decrease productivity^37^. The roots actively seek to absorb increased amounts of water as they expand, thereby enabling plants to adjust and reduce water loss through stomatal closure during periods of water scarcity^38^. Common signs of drought stress in plants comprise leaf curling, stunted growth, yellowing foliage, leaf burning, and irreversible wilting^39^.

### Proline

Proline, an osmotic protector, facilitates plant growth under stress condition^40^. It not only act as an osmotolerant, but also act as a nutritional source i.e., K^+^, Ca^+^, P and N^41,42^. Furthermore, stabilization of mitochondrial electron transport complex, proteins, membranes and enzymes i.e., RUBISCO by exogenous application of proline are also allied factors which played an important role in enhancement of plant growth under stress conditions^43–47^. Additionally, proline has been observed to accumulate in actively dividing meristematic tissues, including the root tip, shoot apex, lateral buds, inflorescence, and germinating seed. It serves as an energy source to sustain these metabolically demanding processes^48,49^.

### Zinc quantum dots

It has been reported that ZnO quantum dots (QDs) facilitated the absorption and accumulation of essential nutrients i.e., Ca, Fe, Mg, Mn, B and Zn. Such improvement in nutrients, enhanced the soluble sugar that resulted in improvement of biomass and quality^15^. In combination with arbuscular mycorrhizae, application of ZQDB significantly enhanced the antioxidant activity i.e., POD, SOD and CAT^12^. POD is involved in scavenging harmful hydrogen peroxide (H_2_O_2_) molecules, which are generated as byproducts of various metabolic processes. It helps to prevent the accumulation of reactive oxygen species (ROS), thereby reducing oxidative damage to cellular components^50^. SOD catalyzes the dismutation of superoxide radicals (O^2−^) into hydrogen peroxide (H_2_O_2_) and molecular oxygen (O_2_) thus alleviate oxidative stress and protects cells from oxidative damage^51^. CAT is another crucial antioxidant enzyme which catalyzes the decomposition of hydrogen peroxide into water and oxygen molecule^52^.

### Biochar

Biochar serves as a valuable tool in alleviating osmotic stress within plants through several key mechanisms^53^. Firstly, its porous structure enables biochar to absorb and retain water, effectively increasing the soil's water holding capacity^54^. This feature becomes particularly beneficial during dry periods, as it ensures a more consistent moisture supply to plant roots, reducing the risk of osmotic stress. Secondly, biochar enhances soil structure by promoting aggregation and reducing compaction, which facilitates better water infiltration and root penetration^55^. Consequently, plants can access water more readily, mitigating the effects of osmotic stress. Moreover, biochar's high cation exchange capacity (CEC) enables it to adsorb and retain essential nutrients in the soil, ensuring their availability to plants even under stressful conditions^56^. This nutrient retention capability is crucial for maintaining plant health and resilience to environmental stressors like osmotic stress. Additionally, biochar stimulates microbial activity in the soil, fostering a healthy soil microbiome that enhances nutrient cycling and promotes shoot and root growth^57^. By supporting these beneficial soil microorganisms, biochar indirectly contributes to the mitigation of osmotic stress by improving nutrient uptake and overall plant vigor^6,14,58^.

## Conclusion

In conclusion, use of 0.4% Zn-quantum dot biochar (ZQDB) with 0.1 mM proline is effective amendment for mitigating negative effects of drought stress. Specifically, 0.4% ZQDB + 0.1 mM Pro can reduce electrolyte leakage and increase the concentration of N, P and K, Such improvements in nutrients concentration and regulation of antioxidants i.e., POD, SOD, APX and MDA by 0.4% ZQDB + 0.1 mM Pro played a vital role in improvement of chili growth under drought stress. Further research is recommended to determine the potential of using 0.4% ZQDB + 0.1 mM Pro as a promising amendment for alleviating drought stress in various other crops, particularly in light of evolving climatic conditions.

## Data Availability

All data generated or analysed during this study are included in this published article.
