# Author Correction: Optimizing chili production in drought stress: combining Zn-quantum dot biochar and proline for improved growth and yield

**DOI:** 10.1038/s41598-024-59029-z

**Published:** 2024-04-09

**Authors:** Misbah Hareem, Subhan Danish, Mahnoor Pervez, Usman Irshad, Shah Fahad, Khadim Dawar, Sulaiman Ali Alharbi, Mohammad Javed Ansari, Rahul Datta

**Affiliations:** 1 Department of Environmental Sciences, Woman University Multan, Multan, Punjab Pakistan; 2 Department of Soil Science, Faculty of Agricultural Sciences and Technology, Bahauddin Zakariya University, Multan, Punjab Pakistan; 3 Department of Zoology, Lahore College for Women University, Lahore, Pakistan; 4 Department of Environmental Sciences, COMSATS University Islamabad Abbottabad Campus, Abbottabad, Pakistan; 5 Department of Agronomy, Abdul Wali Khan University Mardan, Khyber Pakhtunkhwa, 23200 Pakistan; 6 Department of Natural Sciences, Lebanese American University, Byblos, Lebanon; 7 Department of Soil and Environmental Science, The University of Agriculture Peshawar, Peshawar, Pakistan; 8 Department of Botany and Microbiology, College of Science, King Saud University, PO Box -2455, 11451 Riyadh, Saudi Arabia; 9 Department of Botany, Hindu College Moradabad (Mahatma Jyotiba Phule Rohilkhand University Bareilly), Moradabad, India; 10 Department of Geology and Pedology, Faculty of Forestry and Wood Technology, Mendel University in Brno, Zemedelska 1, 61300 Brno, Czech Republic

Correction to: *Scientific Reports* 10.1038/s41598-024-57204-w, published online 19 March 2024

The original version of this Article omitted an affiliation for Shah Fahad. The correct affiliations are listed below.

Department of Agronomy, Abdul Wali Khan University Mardan, Khyber Pakhtunkhwa 23200, Pakistan

Department of Natural Sciences, Lebanese American University, Byblos, Lebanon

The original Article has been corrected.

